# Paradoxical pulmonary artery systolic pressure response with catheter-directed therapies for pulmonary embolism

**DOI:** 10.1016/j.ahjo.2023.100320

**Published:** 2023-09-02

**Authors:** Patrick Ho, Farouk Al-Chami, Mara Caroline, Eric Gnall, Joseph Bonn, Lee Greenspon

**Affiliations:** a Division of Pulmonary & Critical Care Medicine, Lankenau Medical Center, Wynnewood, PA, United States of America; b Division of Internal Medicine, Lankenau Medical Center, Wynnewood, PA, United States of America; c Division of Interventional Cardiology, Lankenau Medical Center, Wynnewood, PA, United States of America; d Division of Interventional Radiology, Lankenau Medical Center, Wynnewood, PA, United States of America

**Keywords:** Pulmonary embolism, Catheter-directed therapy, Pulmonary artery pressure, Ultrasound assisted thrombolysis, Mechanical thrombectomy

## Abstract

**Background:**

Early data on use of catheter-directed therapies (CDT) for treatment of Intermediate or High-Risk pulmonary embolism (PE) show improvement in pulmonary artery systolic pressures (PAsP) and RV/LV ratios. Occasionally a paradoxical rise in PAsP was observed with CDT utilizing ultrasound-assisted thrombolysis (USAT). It is unclear whether this pattern is seen with CDT utilizing mechanical aspiration.

**Objectives:**

To investigate and compare the changes in PAsP between those who underwent CDT with USAT to those with mechanical aspiration.

**Methods:**

A retrospective analysis of those diagnosed with Intermediate or High-Risk PE who underwent CDT using USAT or mechanical aspiration from 7/2013 to 3/2023. The primary outcome was comparison of PAsP changes between the two modalities. Secondary outcomes include length of stay, mortality, and bleeding complications.

**Results:**

A total of 142 patients were analyzed, of which 93 underwent USAT and 49 underwent mechanical thrombectomy. The mechanical thrombectomy group had significantly lower post-intervention PAsP than the USAT group (42.2 ± 13.4 mmHg vs 54.5 ± 15.2 mmHg, *p* < 0.0001) and a greater adjusted mean reduction (−16.5 ± 2.7 vs. −7.7 ± 3.2 mmHg. p < 0.0001). A higher frequency of a paradoxical rise in PAsP was observed in the USAT group (22 % vs 4.1 %, *p* < 0.001).

**Conclusions:**

CDT utilizing mechanical thrombectomy was associated with lower post-interventional PAsP and greater mean negative change compared to USAT. Occasional paradoxical rises in PAsP were observed with both types of CDT, but they were more frequent with USAT. Hemodynamic monitoring should be considered after CDT.

**Condensed unstructured abstract:**

We report a retrospective comparison of changes to pulmonary artery systolic pressures (PAsPs) between catheter-directed ultrasound-assisted thrombolysis (USAT) and catheter-directed mechanical thrombectomy in Intermediate and High-Risk pulmonary embolism. Those treated with mechanical thrombectomy compared to USAT had significantly lower post-interventional PAsP (42.2 ± 13.4 mmHg vs 54.5 ± 15.2 mmHg, *p* < 0.0001) and a greater adjusted mean reduction (−16.2 ± 2.7 vs. −7.5 ± 3.2 mmHg, p < 0.0001). A paradoxical rise in PAsP was observed more frequently in the USAT group than the mechanical thrombectomy group (22 % vs 4.1 %, *p* < 0.001).

## Highlights

• CDT improves Pulmonary Artery Systolic Pressures compared to standard anticoagulation in patients with Intermediate Risk PE.
• Catheter-directed mechanical thrombectomy resulted in a more significant reduction in PAsP than Ultrasound Assisted Thrombolysis
• A paradoxical rise in PAsP may be seen post CDT and was more common with USAT
• Transient Vasoconstriction may occur post Catheter based procedures in the Pulmonary circulation.

## 1 Introduction

The treatment of Intermediate and High Risk pulmonary embolism (PE) has evolved with growing data to support the use of catheter-directed therapies (CDT) over historical use of systemic thrombolysis for efficacy and improved safety [[1], [2], [3]]. Initial trials with CDT utilizing ultrasound-assisted thrombolysis (USAT) resulted in improved pulmonary artery systolic pressures (PAsP) at 18–24 h of up to 30 %, which is similar to initial trials utilizing systemic urokinase and tissue plasminogen activator for treatment of PE [2,[4], [5], [6]]. USAT was also able to demonstrate a reduction in angiographically evaluated obstruction index with Miller scores of about 30–70 % in clot burden, which is comparable to that achieved with systemic thrombolysis [7,8]. Interestingly, in a large prospective multicenter registry, a paradoxical rise in PAsP was observed in approximately 10 % of those who underwent USAT [9]. A similar phenomenon occurred in our initial cohort of patients with Intermediate or High-Risk PE treated with USAT despite a significant reduction in clot obstruction as measured by the Miller Score [10]. It is unclear whether this paradoxical rise is seen with other types of CDT. Earlier data using catheter-directed mechanical thrombectomy demonstrated a 23 % reduction in mean pulmonary artery pressure and similar improvements in the right ventricle-to-left ventricle (RV/LV) ratio as compared to USAT [3,11]. However, data directly comparing the two catheter-directed modalities is limited. We retrospectively reviewed our single center experience with CDT utilizing USAT and mechanical thrombectomy and compared the changes in PAsP between them and determined the frequency of paradoxical rise in PAsP after intervention.

## 2 Methods

### 2.1 Study design

This is a retrospective analysis approved by our Institutional Review Board of patients diagnosed with Intermediate or High-Risk PE who underwent CDT with either USAT or mechanical thrombectomy. Catheter-directed USAT was performed with the EkoSonic™ Endovascular System (Boston Scientific, Seattle, WA) and catheter-directed mechanical thrombectomy was performed with the FlowTiever™ System (Inari Medical, Irvine, CA). We analyzed patients from July 2013 to the end of March 2023. In 2019, we introduced catheter-directed mechanical thrombectomy, which has since become our standard practice. Those who were under 18 years of age, had no confirmatory imaging, or had presented with cardiac arrest were excluded from this analysis. Risk assessment of PE was performed on all patients using the Pulmonary Embolism Severity Index (PESI) and European Society of Cardiology mortality risk guidelines. A proposed criteria to consider use of CDT (Table 1) was used at our institution, but the decision to proceed and with which type of CDT to use was made by our Pulmonary Embolism Response Team (PERT). See supplement appendix for USAT and mechanical thrombectomy protocols.

**Table 1:** Criteria to consider catheter directed therapies for pulmonary embolism.

Pulmonary artery thrombus centrally located in right or left pulmonary artery with evidence of compromised distal segments and one or more of the following:
1. High-risk PE with relative or absolute contraindication for systemic thrombolysis.
2. High-risk PE with stability to be transferred to a cardiac catheterization lab within 2 h.
3. High-risk PE following systemic thrombolysis with residual hemodynamic instability.
4. High/Intermediate-risk PE defined as RV/LV ratio > 1.5 and severe RV dysfunction by transthoracic echocardiogram, positive cardiac biomarkers (troponin or BNP), signs of pulmonary hypertension, persistent tachycardia >100 beats per minute, positive lactate >2 mmol/L.
5. High/Intermediate-risk PE with RV/LV ratio > 1.0, positive cardiac biomarkers (troponin or BNP), and proximal deep vein thrombosis.
6. At least intermediate-risk PE with clot-in-transit in right atrium.

PE = pulmonary embolism; RV/LV = right ventricle-to-left ventricle; BNP = B-type natriuretic peptide.

### 2.2 Primary and secondary outcomes

The primary endpoints analyzed were changes in PAsP from catheter-directed intervention between the two modalities and the frequency of observed paradoxical rise in PAsP. PAsPs for both CDT modalities were measured by right heart catheterization at the start of the procedure and at its completion. Secondary outcomes include hospital length of stay, in-hospital mortality, 30-day mortality, and procedural complications.

### 2.3 Statistical analysis

Standard statistics are used to describe baseline characteristics, primary, and secondary outcomes. To compare the results between USAT and mechanical thrombectomy, we used the chi square test of independence and Fisher's exact test for categorical variables and two sample *t*-tests for continuous variables. The PAsP mean difference between pre- and post-CDT was calculated by subtracting the pre-PAsP and post-PAsP. To account for differences between the groups, adjusted mean differences were obtained by building a multivariable linear regression model. The model was built with baseline characteristics that had a *p* < 0.2, then manual backward selection with AIC. The variables that were adjusted for in the final model were body mass index, race, RV/LV ratio, positive troponin, and DVT. The model was used to adjust the mean differences for each CDT intervention. A *p*-value of <0.05 is considered significant, and all analyses were done in Stata 17.0 (Statacorp, LLC, College Station, TX).

## 3 Results

### 3.1 Patient demographics and characteristics

We identified 144 patients diagnosed with Intermediate or High-Risk PE that received CDT with either USAT or mechanical thrombectomy. Two were excluded from the analysis due to having missing data. Of the 142 patients, 93 underwent USAT and 49 underwent mechanical thrombectomy. Patient characteristics are listed in Table 2. There were no significant differences between the two groups except those who underwent mechanical thrombectomy had a greater RV/LV ratio than those who underwent USAT.

**Table 2:** Demographics and patient characteristics.

	Total	USAT	Mechanical Thrombectomy	p-value
N	**142**	**93/142**	**49/142**	
Age, years	62.7 ± 13.8	62.1 ± 14	63.6 ± 13.5	0.503
Female	64 (45.1 %)	45 (48.4 %)	19 (38.8 %)	0.274
Race				0.108
White	69 (48.6 %)	48 (51.6 %)	21 (42.9 %)	
Black	71 (50 %)	45 (48.4 %)	26 (53.1 %)	
Asian	2 (1.4 %)	0 (0 %)	2 (4.1 %)	
Ethnicity				
Non-Hispanic	142 (100 %)	93 (100 %)	49 (100 %)	
Hispanic	0 (0 %)	0 (0 %)	0 (0 %)	
BMI, kg/m^2^	33.4 ± 8.3	34.3 ± 9.1	31.7 ± 6.3	0.07
PESI				0.671
Class I	13 (9.2 %)	10 (10.7 %)	3 (6.1 %)	
Class II	11 (7.8 %)	8 (8.6 %)	3 (6.1 %)	
Class III	42 (29.6 %)	26 (28.0 %)	16 (32.7 %)	
Class IV	37 (25.1 %)	26 (28.0 %)	11 (22.5 %)	
Class V	39 (27.5 %)	23 (24.7 %)	16 (32.7 %)	
Severity risk group				0.76
Intermediate low	1 (0.7 %)	1 (1.1 %)	0 (0)	
Intermediate high	123 (86.6 %)	80 (86.0 %)	43 (87.8 %)	
High	18 (12.7 %)	12 (12.9 %)	6 (12.2 %)	
RV/LV ratio	2.0 ± 0.8	1.9 ± 0.7	2.30 ± 0.8	0.0012
Positive troponin	125 (88.7 %)	79 (85.0 %)	46 (95.8 %)	0.053
DVT	116 (82.9 %)	80 (87.0 %)	36 (75.0 %)	0.075

All values are in mean +/− SD or n (%) unless otherwise indicated.

USAT = Ultrasound-Assisted Thrombolysis; BMI = body mass index; PESI = Pulmonary Embolism Severity Index; RV/LV = Right Ventricle-to-Left Ventricle, DVT = Deep Vein Thrombosis.

### 3.2 Primary outcomes

**Fig. 1:**
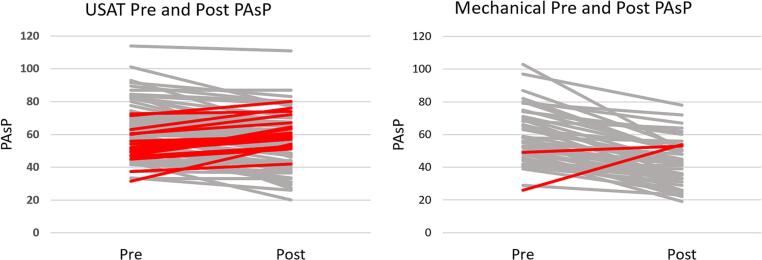
Pulmonary Artery Systolic Pressure Responses to Catheter-Directed Therapies The Pulmonary Artery Systolic Pressure (PAsP) responses pre and post Ultrasound Assisted Thrombolysis (USAT) is compared to the response after Mechanical thrombectomy. The cases in RED represent the cases demonstrating a paradoxical rise in pulmonary artery systolic pressure. In the USAT group (*N* = 93) 22.6 % demonstrated a paradoxical response compared to the Mechanical Thrombectomy group (*N* = 49) 4.1 %. (For interpretation of the references to colour in this figure legend, the reader is referred to the web version of this article.)

Primary outcome data are included in Table 3 and Fig. 1. The initial mean PAsPs between the two groups were not significantly different. However, post-procedural mean PAsP was significantly lower in the mechanical thrombectomy group than the USAT group (42.2 ± 13.4 mmHg vs 54.5 ± 15.2 mmHg, *p* < 0.0001). There also was a significant difference between the mean changes of both groups. Those who underwent mechanical thrombectomy had a greater reduction than those who underwent USAT (−16.2 ± 12.6 mmHg vs. -7.5 ± 12.2 mmHg, *p* = 0.0001), and this reduction remained significant when adjusted for variables (−16.5 ± 2.7 vs. -7.7 ± 3.2 mmHg. *p* < 0.0001). A paradoxical rise in PAsP was more frequently observed in the USAT group compared to the mechanical thrombectomy group (22 % vs 4.1 %, *p* < 0.001).

**Table 3:** Primary outcomes.

	USAT *N* = 93	Mechanical thrombectomy *N* = 49	p-Value
Pre-CDT PAsP, mmHg	62.1 ± 15.4	58.3 ± 16.4	0.183
Post-CDT PAsP, mmHg	54.5 ± 15.2	42.2 ± 13.4	<0.0001
Mean change, mmHg	−7.5 ± 12.2	−16.2 ± 12.6	0.0001
Adjusted mean change^a^, mmHg	−7.7 ± 3.2	−16.5 ± 2.7	<0.0001
Paradoxical rise in PAsP	21 (22.6 %)	2 (4.1 %)	0.008

All values are in mean +/− SD or n (%) unless otherwise indicated.

USAT = Ultrasound-Assisted Thrombolysis; CDT = Catheter-directed therapy; PAsP = Pulmonary Artery Systolic Pressure.

a Adjusted for BMI, race, RV/LV, positive troponin, deep vein thrombosis.

### 3.3 Secondary outcomes/safety data

Secondary outcomes are included in Table 4. The median hospital length of stay was significantly longer in the USAT group than the mechanical thrombectomy group. There were no significant differences between in-hospital and 30-day mortality rates between the two groups. There were no significant differences in bleeding complications between the two groups, but they each had one patient who experienced a significant bleeding complication directly related to the procedure. Both incidents were considered Bleeding Academic Research Consortium type 3 and required transfusions [12]. In patients with a paradoxical rise in PAsP with available follow up echocardiography, no evidence of chronic thromboembolic pulmonary hypertension or persistent right ventricular dysfunction was seen.

**Table 4:** Secondary outcomes.

	USAT	Mechanical thrombectomy	p-Value
Length of stay (median, IQR), days	6 (5–8)	4 (3–7)	<0.0001
In-hospital mortality	3 (3.2 %)	0 (0)	0.204
30-day mortality	3 (3.2 %)	0 (0)	0.204
Bleeding complications	1 (1.1 %)	1 (2.0 %)	1.0
Hemoptysis	0	1	
Catheter-site	1	0	

All values are in n (%) unless otherwise indicated.

USAT = Ultrasound-Assisted Thrombolysis; LOS = Length of Stay; IQR = Interquartile Range.

## 4 Discussion

The benefits of CDT in patients with Intermediate and High-Risk PE in prior studies have generally relied on evaluation of changes in RV/LV ratios at 48 h. Several studies of CDT that had measured changes in PAsP showed significant improvements [2,4,9,13]. Our results are in line with these reports. We had found that both CDT utilizing USAT and mechanical thrombectomy resulted in improvement of PAsP. However, our analysis revealed an even greater reduction of PAsP in those treated with mechanical thrombectomy than those treated with USAT. In addition, a paradoxical rise in PAsP was observed in both groups. However, the frequency of this paradoxical rise was significantly higher in the USAT group and was not as readily observed in the mechanical thrombectomy group.

The observed greater reduction in mean PAsP from catheter-directed mechanical thrombectomy compared to USAT could have several explanations. The paradoxical rise in PAsP of 22 % in the USAT group reduced the overall response calculated for this group of patients. The absolute clot burden as estimated by Miller scores was not done for this study. Pulmonary vascular resistance (PVR) has an exponential relationship with the degree of clot obstruction when there is >60 % clot obstruction [7]. Those patients with the greatest clot burden will therefore have the greatest change in PAsP, whereas those patients with <50 % clot burden may have little change in PAsP. This may explain why thrombolysis or mechanical thrombus removal does not always correlate with the PAsP response. Our USAT protocol delivered 0.5 mg of tissue plasminogen activator per hour per catheter for up to 24 h. Our prior studies using this approach and calculating Miller scores showed >50 % reduction in clot burden. This should have been equivalent to that achieved by mechanical thrombectomy.

The mechanism behind the paradoxical rise in PAsP is unclear. It could be related to increasing right-sided cardiac output through a fixed, resistant vascular bed post clot removal. In addition, clot removal could also cause the release of vasoactive compounds like endothelins and thromboxane A2. Hemolysis and free hemoglobin could potentially reduce the availability of nitric oxide. Microembolization of clot into peripheral artery beds could be an additional factor [14]. Perhaps the sudden increase in pulmonary vascular flow induces vasoconstriction in some patients. Of interest, a recent case report showed a similar paradoxical transient increase in pulmonary artery pressures in patients undergoing balloon pulmonary angioplasty for chronic thromboembolic pulmonary hypertension [15]. We suspect that transient vasoconstriction is the most likely explanation and that this may be seen more frequently with USAT since the catheters for thrombolysis delivery often stay in the pulmonary circulation for 6–20 h .Since paradoxical rises of PAsP were seen with both CDT, this phenomenon could likely occur with all forms of CDT. Therefore, future cases utilizing any type of CDT should be aware of this potential response.

A strength of this report is that this is one of the few studies that directly compared two catheter-directed therapies to each other for the treatment of Intermediate and High-Risk PE from one center. In addition, we highlight the occasional paradoxical rise in PAsP that can occur after CDT, which had not been discussed in prior studies. A rapid acute rise in PAsP can potentially cause sudden patient deterioration, especially with existing right heart dysfunction. As such, this phenomenon could have clinical implications, and post-procedural hemodynamic monitoring has become our standard.

There are also limitations to this report. We did not measure clot burden by Miller score pre and post procedure. We did not perform full hemodynamic measures of cardiac output and calculated PVR in a majority of patients to better evaluate the responses. Finally, given that the decision to proceed with CDT was determined by PERT, patient selection could have been biased and the patients of this study might only be reflective of a certain few.

## 5 Conclusion

Both CDT utilizing USAT and mechanical thrombectomy resulted in improved pulmonary artery systolic pressures, though the degree of improvement was significantly greater in those treated with mechanical thrombectomy. Furthermore, we raise awareness that a paradoxical rise in PAsP can be seen with CDT, which appeared to occur more frequently in those treated with USAT than those treated with mechanical thrombectomy. Whether this paradoxical rise is clinically impactful or has implications is yet to be determined, but it could influence a patient's recovery after intervention. Monitoring hemodynamics post-procedure is the only way to alert physicians that this paradoxical response has occurred.

## Declaration of competing interest

The authors declare the following financial interests/personal relationships which may be considered as potential competing interests: Lee Greenspon reports a relationship with Inari Medical that includes: funding grants. Lee Greenspon reports a relationship with 10.13039/100008497Boston Scientific Corp. that includes: funding grants. Joseph Bonn reports a relationship with Boston Scientific Corp that includes: funding grants.

## Appendix A Supplementary data

Supplementary material Image 1
