# Supplementary material for: Paradoxical pulmonary artery systolic pressure response with catheter-directed therapies for pulmonary embolism
Source: Am Heart J Plus. 2023 Sep 2;34:100320. doi: 10.1016/j.ahjo.2023.100320 (PMC10946050; doi:10.1016/j.ahjo.2023.100320)
Supplement: Supplementary file 1 — Supplementary material [file mmc1.docx]

**Paradoxical Pulmonary Artery Systolic Pressure Response with Catheter-Directed Therapies for Pulmonary Embolism**

(Running Title: Paradoxical Pulmonary Artery Pressure Response with Catheter Directed Therapies for Pulmonary Embolism)

Patrick Ho, DO^a^, Farouk Al-Chami, MD^b^, Mara Caroline, MD^c^, Eric Gnall, DO, FACC^c^, Joseph Bonn MD^d^, Lee Greenspon, MD, FCCP^a^

**Supplemental Material**:

**Catheter-directed Ultrasound Assisted Thrombolysis ( USAT ) was performed with the EkoSonic™ Endovascular System (Boston Scientific, Seattle, WA)**

**Equipment:**

6F sheath(s)for Ultrasound established femoral vein vascular access.

Catheter and wire to access the pulmonary artery:

         - H1H and Glidewire with pigtail catheter for femoral approach.

         - Cobra and Glidewire or Swan-Ganz for IJV approach.

260cm Rosen for all exchanges (1.5J tip reduces perforation risk).

5F pigtail for pulmonary arteriograms.

Power injector loaded.

EKOS**^®^** kit(s): choose infusion length and working length per patient.

         - cover electrical connectors with Tegaderm to keep dry.

Alteplase (tPA): see dosing below; order from Pharmacy before procedure begins.

**Technique:**

1. Access main pulmonary artery; measure main PA mean PAP and MVO2.
2. Access pulmonary artery of interest.
3. Exchange over Rosen wire for 5F pigtail.
4. Measure unilateral pulmonary artery pressures (systolic/diastolic, mean).
5. Power-inject pulmonary arteriogram(s) in best diagnostic projection.
6. Direct Rosen wire into pulmonary artery segment with greatest thrombus burden.
7. Exchange over Rosen wire for EKOS**^®^** multi-sidehole catheter of appropriate length.
8. Position EKOS**^®^** catheter into thrombus.
9. Advance EKOS**^®^** ultrasonic core into catheter, luer lock in position.
10. Secure and dress sheath; mark EKOS**^®^** catheter at sheath valve; secure catheter.
11. Connect coolant port to NS at 35ml/hour.
12. Sheath sidearm may be used for IV systemic heparin; other infusions/meds.

**Bilateral EKOS^®^ tPA dosing:**

10mg tPA in 500ml NS for each catheter (two identical bags).

each bag to infuse at maximum of 25ml/hour.

         = 0.5mg tPA per hour per catheter, or total 1.0mg tPA per hour.

**Unilateral EKOS^®^ tPA dosing:**

20mg tPA in 500ml NS.

infuse at 1.0mg tPA per hour =25ml/hour. (max recommended flow rate is 35 ml/hr)

**Mechanical Thrombectomy Procedure:**

**FlowTiever™ System (Inari Medical, Irvine, CA)**

We first gain ultrasound access to the common femoral vein. Following this over a stiff wire we place a delivery sheath.

Usually a therapeutic ACT is achieved. Next a balloon tipped catheter is directed into the right pulmonary artery if possible

And pressure measurements Cardiac output are determined. Following this weeks change the balloon tip catheter for six French pigtail catheter And a pulmonary angiogram is performed. Through the pigtail catheter we loop a 6 cm floppy tipped super stiff amplatz wire to avoid wire perforation. Over Over this wire we deliver the 24 French flowtriever. Thrombus extraction is performed repeat pulmonary angiogram is performed. Following this we either retract flowtriever and redirect the wire to the left or are use a pigtail catheter to perform the same maneuver. On the left side we frequently use the 20 curve flowtriever in conjunction with the 24 French  flowtriever. Repeat pulmonary angiogram is performed after thrombus extraction. Re-measurements of cardiac output and PA pressures are performed via the flowtriever catheter. Access site was either pre-closed, or over sewn in a horizontal mattress suture using heavy silk

I forgot one important thing prior to beginning the procedure a radial a line is Is placed for hemodynamic monitoring.
